# *RORA* and posttraumatic stress trajectories: main effects and interactions with childhood physical abuse history

**DOI:** 10.1002/brb3.323

**Published:** 2015-03-08

**Authors:** Sarah R Lowe, Jacquelyn L Meyers, Sandro Galea, Allison E Aiello, Monica Uddin, Derek E Wildman, Karestan C Koenen

**Affiliations:** 1 Department of Epidemiology, Mailman School of Public Health, Columbia UniversityNew York, New York; 2 Department of Epidemiology, Boston University School of Public HealthBoston, Massachusetts; 3 Department of Epidemiology, Gillings School of Global Public Health, University of North CarolinaChapel Hill, North Carolina; 4 Department of Psychology, University of Illinois Urbana-ChampaignChampaign, Illinois; 5 Institute for Genomic Biology, University of Illinois Urbana-ChampaignUrbana, Illinois; 6 Depratment of Molecular and Integrative Physiology, University of Illinois Urbana-ChampaignUrbana, Illinois

**Keywords:** Childhood physical abuse, gene-environment interaction, longitudinal survey, posttraumatic stress disorders, retinoid-related orphan receptor-alpha

## Abstract

**Background:**

Longitudinal studies of posttraumatic stress (PTS) have documented environmental factors as predictors of trajectories of higher, versus lower, symptoms, among them experiences of childhood physical abuse. Although it is now well-accepted that genes and environments jointly shape the risk of PTS, no published studies have investigated genes, or gene-by-environment interactions (GxEs), as predictors of PTS trajectories. The purpose of this study was to fill this gap.

**Methods and Materials:**

We examined associations between variants of the retinoid-related orphan receptor alpha (*RORA*) gene and trajectory membership among a sample of predominantly non-Hispanic Black urban adults (*N *=* *473). The RORA gene was selected based on its association with posttraumatic stress disorder (PTSD) in the first PTSD genome wide association study. Additionally, we explored GxEs between *RORA* variants and childhood physical abuse history.

**Results:**

We found that the minor allele of the *RORA* SNP rs893290 was a significant predictor of membership in a trajectory of consistently high PTS, relatively to a trajectory of consistently low PTS. Additionally, the GxE of rs893290 with childhood physical abuse was significant. Decomposition of the interaction showed that minor allele frequency was more strongly associated with membership in consistently high or decreasing PTS trajectories, relative to a consistently low PTS trajectory, among participants with higher levels of childhood physical abuse.

**Conclusion:**

The results of the study provide preliminary evidence that variation in the *RORA* gene is associated with membership in trajectories of higher PTS and that these associations are stronger among persons exposed to childhood physical abuse. Replication and analysis of functional data are needed to further our understanding of how *RORA* relates to PTS trajectories.

## Introduction

Posttraumatic stress disorder (PTSD) is a debilitating mental disorder characterized by traumatic intrusions, experiential and behavioral avoidance, emotional numbing, and hyperarousal (American Psychological Association 2013). The lifetime prevalence of PTSD in the United States general population has been estimated at 7.3%, and 9.1% among persons who have experienced traumatic events (Roberts et al. 2011). There is variation in the course of posttraumatic stress (PTS) symptoms among those who meet diagnostic criteria for PTSD, such that some persons have symptoms that follow a chronic course, whereas others experience symptom recovery or a relapsing and remitting symptom pattern (Norris et al. 2009). Furthermore, among persons who do not initially develop PTSD, some continue to experience few or no symptoms, whereas others exhibit a delayed response, meeting criteria for the disorder months or years after the traumatic event (Norris et al. 2009). Given these observations, recent studies have explored longitudinal trajectories of PTS, documenting the relative frequency and predictors of different symptom patterns over time (Norris et al. 2009; Steenkamp et al. 2012; Lowe et al. 2014).

Posttraumatic stress trajectory studies have identified environmental factors as predictive of trajectories of higher, versus lower, symptoms, among them characteristics associated with socioeconomic disadvantage, more extensive trauma exposure, and fewer social resources (Dickstein et al. 2010; Lowe et al. 2014). One environmental factor that seems to be an especially robust predictor of higher PTS trajectories is exposure to early life stress, including experiences of childhood physical abuse (Dickstein et al. 2010; Lowe et al. 2014). This is consistent with the concept of stress sensitization, which posits that early trauma exposure alters stress response systems such that they are primed to respond in ways that increase risk for PTSD after later exposure (McLaughlin et al. 2010; Yehuda et al. 2010).

In contrast with the evidence of environmental predictors of PTS trajectories, no published study to our knowledge has explored associations between genes and PTS trajectory membership. One gene that could relate to PTS trajectories is the retinoid-related orphan receptor alpha (*RORA*) gene, located on the long arm of chromosome 15 (15q22.2). In the first genome wide association study of a cross-sectional lifetime PTSD diagnosis, the rs8042149 single nucleotide polymorphism (SNP) within the *RORA* gene (base pair [bp] position 61,124,953; alleles, minor/major: G/A; minor allele frequency [MAF] = 0.18) was the only SNP to reach the established level for genome-wide significance (*P *<* *5 × 10^−8^) in the discovery sample of non-Hispanic White United States military veterans (OR = 2.5, *P *=* *2.5 × 10^−8^) (Logue et al. 2010) (The variant positions and MAFs listed are from the SNP database [dbSNP; http://www.ncbi.nlm.nih.gov/projects/SNP]). Variant positions are from the 1000 Genomes project, and MAFs are from the HapMap-YRI sample). Notably, only nine other SNPs across the genome reached the established level for suggestive evidence of association (*P *<* *5 × 10^−5^), including four other *RORA* SNPs. Among the five variants outside of the *RORA* gene, the strongest suggestive association had a *P*-value of 4.3 × 10^−6^. Although the one genome-wide significant SNP (rs8042149) was not significant in the study's replication sample, other SNPs within *RORA* were significant predictors of PTSD, the strongest predictors being rs16942660 (bp position 60,801,066; alleles, minor/major: T/C; MAF = 0.12) and rs893290 (bp position 61,438,153 15pter; alleles, minor/major: G/A; MAF = 0.11). A subsequent study of European Americans replicated the finding from the GWAS discovery sample, further supporting the links between *RORA* and PTSD (Amstadter et al. 2013).

Although *RORA* has only been recently linked to PTSD, prior studies have linked the gene to other forms of psychopathology, including bipolar disorder (Le-Niculescu et al. 2009), major depression (Garrilock et al. 2010), and attention deficit hyperactivity disorder (Neale et al. 2008), as well as to neurodevelopmental disorders, such as autism (Nguyen et al. 2010). To provide insight into the role of *RORA* in these potentially overlapping disorders, a recent study on the aforementioned veteran sample examined associations between *RORA* SNPs and factors indicative of three broad dimensions of psychiatric illness: fear, distress, and externalizing factors (Miller et al. 2013). The *RORA* SNPs were predictive of the fear factor, which included symptoms of panic, agoraphobia, specific phobia, and obsessive compulsive disorder, but not the distress or externalizing factors. PTSD was excluded from this conceptualization, given that its association with *RORA* had been previously documented in the sample. However, PTSD also has been conceptualized as a fear disorder (Amstadter et al. 2009).

No functional data linking *RORA* to PTSD has been published and therefore the biological mechanisms remain unknown. It is likely, however, that the processes underlying this association are complex and play out over time. An investigation of the relationship between *RORA* SNPs and PTS trajectories could provide insight into the temporal nature of this relationship, for example shedding light on the short-versus longer term genetic vulnerability for PTSD (Koenen et al. 2008).

It is also likely that PTS trajectory membership is shaped by the interplay of environmental factors and genetic factors (Koenen et al. 2008; Skelton et al. 2012). Previous research has documented gene-by-environment interactions (GxEs) for PTSD and other fear-related disorders (Stein et al. 2002; Lau et al. 2007; Hicks et al. 2009). including GxEs between various forms of early life stress, including childhood physical abuse, and other PTSD candidate genes (Binder et al. 2008; Xie et al. 2009; Nugent et al. 2011). Early life stress represents an ideal environmental exposure to include in GxE studies, given that such experiences are strongly associated with PTSD in adulthood, yet there remains vast heterogeneity in the psychological functioning of adults who have endured them. Such heterogeneity indicates the possibility of a potential GxE (Koenen et al. 2008). Because significant genome-wide associations have been identified between *RORA* variants and PTSD, *RORA* is a suitable candidate gene to explore in GxEs with early life stress, despite the lack of data regarding the mechanisms underlying these associations (Nugent et al. 2011).

Notably, several methodological considerations about GxE analyses have been raised in recent literature (Koenen et al. 2008; Dick 2011; Duncan and Keller 2011; Nugent et al. 2011). Although a comprehensive review of these considerations is beyond the scope of this article, we highlight six of them here. First, a lack of statistical power in GxE studies, particularly those with small samples, can limit the ability both to detect true effects as well as increase the likelihood of false discovery findings (Duncan and Keller 2011). Trajectory analysis has been proposed as one way to address the issue of statistical power in GxE studies (McArdle and Prescott 2010). However, no published study has investigated the role of GxEs in influencing PTS trajectory membership. Second, the use of dichotomous indicators of environmental exposures further contributes to low power due to lack of variance in interaction terms and, as such, continuous indicators are recommended (Duncan and Keller 2011). Third, GxE studies with early life stress as the environmental factor have included a vast array of events that vary in nature, timing, and severity (Nugent et al. 2011). These sources of variation could certainly influence the presence of a GxE and, accordingly, it is essential for researchers to specify that environmental exposure that is being measured and attend to the limitations of their approach. Fourth, population stratification, or variation in allele frequency among participants of different genetic ancestries, could lead to spurious GxE findings and, as such, it is important to account for ancestry in statistical analyses (Nugent et al. 2011). Fifth, age and gender should also be considered, as these factors have been found to influence associations between genetic and environmental factors and mental health outcomes (Nugent et al. 2011). Finally, researchers should assess the potential for gene-environment correlation (rGE), or associations between genetic variants and environmental exposures, which reflect the potential for individuals to influence their environment and complicate the interpretation of GxE findings (Moffitt et al. 2005; Dick 2011).

## Current Study

This study is the first to explore the role of genes and GxEs in predicting PTS trajectory membership. We examined associations between *RORA* SNPs, as well as GxEs between *RORA* SNPs and childhood physical abuse, and PTS trajectories among a sample of urban adults. We focus on *RORA* because variants of this gene reached genome wide significance in a prior study, and selected two SNPs that were previously shown to predict PTSD in our sample, rs16942669 and rs893290 (Logue et al. 2010). A continuous indicator of childhood physical abuse prior to age 18 was selected as the environmental exposure, given both the heterogeneity in responses to early life stress and prior evidence that genetic variants moderate the influence of early life stress on PTSD. We acknowledge from the outset two key limitations of our environmental measure: first, it did not include other forms of early life stress (e.g., childhood sexual and emotional abuse), as well as stressors and traumatic events in adulthood, that tend to co-vary with childhood physical abuse and could also account for the presence of GxE (Whitfield et al. 2003; Clemmons et al. 2007; Nugent et al. 2011); second, it lacked specificity in that the developmental timing of childhood physical abuse experiences was not assessed. In our analyses, we controlled for ancestry markers, age and gender in our analysis, and considered the ways in which rGE could influence our findings.

## Methods and Materials

### Participants

Data were from the genetic sample of the Detroit Neighborhood Health Study (DNHS), a longitudinal population-based study of predominantly non-Hispanic Black adults (18 years or older) living in Detroit, Michigan (*N *=* *778) (Uddin et al. 2010). Analyses were conducted on participants from the genetic sample who (1) reported at least one lifetime trauma and completed the PTS measure in the first wave of data collection and could therefore be part of the trajectory analysis (*N *=* *650; 83.5% of the full genetic sample), and (2) had complete data on childhood physical abuse, age, and gender (*N *=* *473; 60.8% of the full genetic sample; 72.8% retention). Detailed information on the construction of the DNHS sample has been published elsewhere (Uddin et al. 2010). Wave 1 (W1) of the study was conducted between 2008 and 2009, and Wave 2 (W2) and Wave 3 (W3) approximately one and two years thereafter respectively. At each wave, participants completed a structured telephone survey, which lasted an average of 40 min and included a measure of lifetime PTS. Informed consent was obtained at the beginning of each survey, and participants were offered $25 for their participation in each interview.

### DNA samples and genotyping

Participants in the genetic sample provided blood specimens by way of venipuncture (*n *=* *688) or saliva (*n *= 125). Informed consent was obtained prior to DNA collection, and participants were offered $25 for providing samples. Further details on the DNA collection have been published previously (Uddin et al. 2010). Briefly, DNA samples were genotyped using the HumanOmniExpress Bead-Chips (Illumina, San Diego, CA). Of the 813 collected samples, 34 were removed during quality control procedures, yielding a final genetic sample of 778 participants. Both *RORA* SNPs of interest (rs16942669 and rs893290) passed quality control filters (call rate >95%, MAF > 0.01, Hardy-Weinberg disequilibrium *P *>* *1 × 10^−6^).

In PLINK, Multi-Dimensional Scaling (MDS) analysis of genome-wide identity-by-state was used to determine ancestry using the 688,890 SNPs that passed the aforementioned quality control filters. As described previously (Meyers et al. 2013), two principal components from the MDS analysis identified clearly separated clusters that correlated with self-reported ethnicity identification. These components were included as ancestry markers in the analysis.

### Measures

#### Posttraumatic stress

At W1, participants completed a 20-item trauma inventory, wherein they indicated whether they had experienced 19 traumatic events in their lifetime, as well as an additional item allowing participants to indicate whether they had experienced another traumatic event not on the inventory (Breslau et al. 1998). After completing the inventory, participants were asked to indicate which of the lifetime traumatic events was the “worst.”

Posttraumatic stress related to the “worst” lifetime trauma was assessed using items drawn from the PTSD Checklist-Civilian Version (Weathers and Ford 1996). Participants indicated the degree to which they had been bothered by each symptom as a result of the event from 1 = *Not at all* to 5 = *Extremely*. The inventory included 17 items, representing criteria B (re-experiencing: five items, e.g., “repeated, disturbing thoughts or memories about the event”), C (avoidance: two items, e.g., “avoiding activities or situations because they reminded you of the stressful experience”; and emotional numbing: five items, e.g., “loss of interest in things you used to enjoy”), and D (hyperarousal: five items, e.g., “trouble falling and staying asleep”) from the DSM-IV (American Psychological Association 1994). At each assessment, participants responded based their “worst” W1 event. At W1, participants indicated the extent they were *ever* bothered by each symptom, and at W2 and W3, the extent they were bothered since the prior interview. Responses were summed to yield a total severity score ranging from 17 to 85. A cut-off score of 44 has been established for normative samples, such that scores exceeding this threshold are indicative of probable PTSD (Blanchard et al. 1996; National Center for PTSD[Bibr b39]). Based on this criteria, 26.0% of participants had probable PTSD at W1, 19.9% at W2, and 21.7% at W3. The internal consistency of the scale in this study ranged from Cronbach's alpha of 0.93 to 0.97.

Clinical in-person interviews with a random subsample of 51 DNHS participants supported the validity of DSM-IV PTSD diagnoses based on meeting criteria on the intrusion, avoidance, and hyperarousal items of the scale, as well as additional items assessing feelings of helplessness and hopelessness during the event, duration of symptoms, and significant distress and functional impairment, relative to the Clinician-Administered PTSD Scale for DSM-IV (Blake et al. 1995) (sensitivity = 0.24, specificity = 0.97, positive predictive value = 0.80, negative predictive value = 0.72, and area under the receiver-operating characteristic [ROC] curve = 0.76).

#### Childhood physical abuse

In addition to reporting on the traumatic events described above, participants completed six items from the physical assault subscale of the Conflicts Tactics Scale (CTS) (Straus and Hamby 1995) assessing the frequency of physical abuse by a parent, stepparent, or adult guardian that occurred before age 18. The CTS is a widely used measure with strong evidence of validity and reliability (Straus 1990). Items (e.g., “spank you for discipline,” “push, grab, or shove you”) were rated from 0 (*Never*) to 4 (*More than a few times*), and childhood physical abuse severity scores were computed as the sum of all items. In the current study, internal consistency of the CTS physical assault subscale was good (Cronbach's alpha = 0.79).

### Data analysis

Prior to testing the study aims, a series of descriptive analyses were conducted. First, Bonferroni-corrected independent-samples *t*-tests and chi-square tests assessed differences between the 473 included participants and 305 participants who were dropped based on our inclusion criteria. Second, we tested for gene-environment correlation between each *RORA* SNP and childhood physical abuse using Spearman's *rGE*. This set of analyses was conducted in SPSS 20.0 (IBM Corp 2011).

Subsequently, PTS trajectories were derived in Mplus 7.1 (Muthén and Muthén 1998–2012) through latent class growth analysis (LCGA), a group-based mixture modeling procedure. Group-based modeling is contrasted with traditional growth curve modeling (GCM), wherein an average trajectory is estimated for the full sample and predictor variables are entered to explain residual variance in intercept and slope terms (Jung and Wikrama 2008; Andruff et al. 2009; Nagin 2009). A drawback in GCM is that it is assumed that participants come from the same population and that therefore a single growth trajectory can be approximated for the entire sample (Jung and Wikrama 2008). These assumptions are at odds with theoretical frameworks and research findings showing subpopulations with varying patterns of growth (Jung and Wikrama 2008). Group-based mixture models overcome this limitation by identifying latent, or unobserved, classes of trajectories within a sample (Nagin 2009). Unlike a general Growth Mixture Modeling (GMM) approach, LCGA imposes the restriction that intercept and growth terms within each trajectory are constant (i.e., the variance of these terms is fixed at zero) (Jung and Wikrama 2008). The advantage of these restrictions is that models are more easily specified; for example, quadratic growth terms can be estimated with only three waves of data (Jung and Wikrama 2008). Additionally, LCGA fit the aims of the study, which was to explore predictors of trajectory membership, rather than variance in growth terms within trajectories. We ran five LCGA models that included linear and quadratic and different numbers of classes, ranging from 2 to 6. As there is no definitive test for determining which model best represents the data, we followed recommended practices by taking into account statistical criteria, the substantive meaning of each solution, theory, and parsimony (Nylund et al. 2007; Masyn 2013; Berlin et al. 2014). Statistical criteria included the Bayesian information criterion (BIC), and adjusted BIC, with lower values indicating better fit; entropy and average posterior probabilities, with higher values indicating better fit; and the Lo-Mendell-Rubin adjusted likelihood ratio test (LMR-LRT), which examines whether a model with *k* classes has significantly better fit than a model with *k *− 1 classes (Jung and Wikrama 2008; Andruff et al. 2009). After the model that best represented that data were selected, probabilities of membership in each class (continuous variables; range: 0–1) and most likely class membership (categorical variable) for each participant were exported and merged into the study dataset.

Analyses to fulfill the study aims were then tested in Mplus 7.1. Rather than computing a standard multinomial regression models predicting participants’ most likely class membership, we used a statistical procedure that takes into account classification uncertainty in the LCGA model by incorporating class membership probabilities into the analysis (Block et al. 2004; Vermunt 2010; Asparouhov and Múthen 2013). This procedure has been found to reduce systemic bias in which associations between covariates and class membership are underestimated, and produces coefficients that are analogous to those from standard multinomial regression models (Vermunt 2010; Asparouhov and Múthen 2013). The first set of predictive models tested main effects of each *RORA* SNP and controlled for female gender and age, as well as the two ancestry principal components. In the second set of predictive models, the main effect of childhood physical abuse and interaction between childhood physical abuse and each *RORA* SNP were entered. Models were run with different referent groups to assess associations between predictors and the likelihood of membership in each trajectory, relative to each other trajectory. In the Results, we present nominally significant results (*P *<* *0.05) results with a Bonferroni correction for the number of SNPs being analyzed (*α*/2; *P *<* *0.025), which was consistent with the approach used in a prior study assessing genetic variants and GxEs as predictors of mental health trajectories (Latendresse et al. 2011). To provide insight into the nature of significant main and interaction effects, cross-tabulations of the categorical variable indicating participants’ most likely trajectory membership by genotype were computed in SPSS 20.0 for the full sample and stratified by high versus low levels of childhood physical abuse (derived through median split).

## Results

### Descriptive statistics

Table 1 lists descriptive data on all study variables for the 473 participants in the analytic sample. The majority were female (57.5%) and identified as Black or African American (83.1%). On average, participants were 53.35 years old (SD = 15.58). Compared to participants in the analytic sample, those dropped based on our inclusion criteria reported significantly lower childhood physical abuse (*t*_113_ = 4.74, *P *<* *0.001; Table 2009). Gene-by-environment correlations (*rGE*s) between the two *RORA* SNPs and childhood physical abuse were both nonsignificant (rs16942669: rGE = −0.07, *P *=* *0.11; rs893290: rGE* *= 0.05, *P *=* *0.31).

**Table 1:** Descriptive data for the DNHS genetic sample and analytic subsample

	Genetic full sample (*N *=* *778)		Analytic sample (*N *=* *473)	
	M (SD)/%	*N*	M (SD)/%	*N*
Wave 1 PTS	35.53 (15.80)	577	35.02 (15.68)	473
Wave 2 PTS	28.30 (12.04)	461	32.26 (14.37)	276
Wave 3 PTS	30.22 (13.18)	292	32.68 (15.26)	217
Childhood physical abuse	3.35 (3.75)	545	3.59 (3.81)^1^	473
rs16942660 (A)		777		473
0	78.0%		79.5%	
1	20.5%		19.0%	
2	1.5%		1.5%	
rs893290 (C)		776		473
0	80.3%		80.3%	
1	18.3%		18.8%	
2	1.4%		0.8%	
Female	57.7%	778	57.5%	473
Age	52.65 (16.38)	638	53.35 (15.58)	473
Black or African American	82.5%	778	83.1%	473
Ancestry-principal component 1	0.00 (0.05)	778	0.00 (0.05)	473
Ancestry-principal component 2	0.00 (0.01)	778	0.00 (0.01)	473

Minor alleles for *RORA* SNPs are listed in parentheses. Genotype designation (0, 1, 2) reflects copies of the minor allele.

1 Significant difference between the Genetic Full Sample and Analytic Subsample (assessed via Bonferroni-corrected independent-samples *t*-tests and chi-square tests).

### Posttraumatic stress trajectories

Table 2 lists the results of the five LCGA models. The model with four trajectories was chosen as the best representation of the data. Compared to the three-class model, the four-class model had lower BIC and adjusted BIC values, higher entropy and mean posterior probability, and a significant LMR-LRT test. The five-class model had a lower BIC and adjusted BIC, as well as higher entropy and mean posterior probability; however, the LMR-LRT was nonsignificant, indicating that it did not have significantly better fit than the four-class model. Additionally, the trajectories in the four-class model were consistent with those from an LCGA using a larger sample of DNHS participants (Lowe et al. 2014).

**Table 2:** Fit statistics for the latent class models (*N* = 473)

Classes	BIC	Adj BIC	Entropy	Mean posterior probability (SD; Range)	LMR-LRT
2	7819.86	7788.12	0.81	0.94 (0.03; 0.92–0.96)	*P *<* *0.001
3	7802.06	7757.63	0.74	0.84 (0.07; 0.78–0.91)	*P *=* *0.32
**4**	**7740.24**	**7683.11**	**0.83**	**0.87 (0.05; 0.84–0.94)**	***P *****=***** *****0.03**
5	7733.52	7663.69	0.84	0.88 (0.07; 0.80–0.94)	*P *=* *0.61
6	7735.93	7653.41	0.81	0.86 (0.06; 0.77–0.92)	*P *=* *0.19

BIC, Bayesian Information Criterion; Adj, Adjusted; SD, Standard Deviation; LMR-LRT, Lo-Mendell-Rubin Likelihood Ratio Test.

For clarity, the model selected as the best representation of the data is listed in boldface.

**Figure 1:**
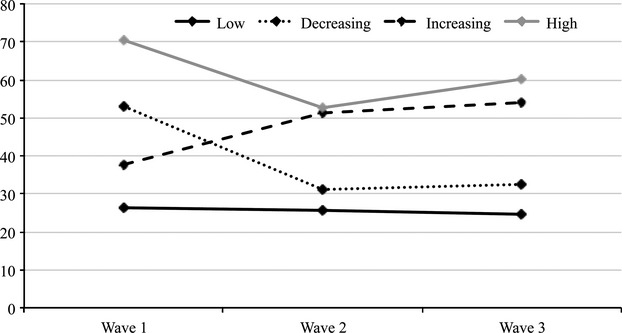
Graph of Estimated Means on the PTSD Checklist-Civilian Version (PCL-C) for the Four Latent Class Trajectories (*N *=* *473).

The four trajectories were given names based on observed patterns of growth and that were consistent with the aforementioned prior DNHS LCGA study (Lowe et al. 2014). Estimated means for each trajectory are shown in Fig.1, and descriptive data are provided in Table 3. The majority of participants were in the *low* trajectory (*n *=* *322, 68.1%). The *low* trajectory evidenced neither significant linear nor quadratic growth, and on average, participants’ PTS never exceeded the cut-off for probable PTSD. The second most prevalent trajectory was labeled *decreasing* (*n *=* *82, 17.3%), and was defined by significant linear and quadratic growth. On average, participants in the *decreasing* trajectory exceeded the cut-off for probable PTSD at W1, and were below the cut-off at W2 and W3. Thirty-eight participants (8.0%) were in the *increasing* trajectory, defined by significant linear growth, and had PTS scores below the cut-off at W1 and above the cut-off at W2 and W3. Lastly, the *high* trajectory (*n *=* *31, 6.6%) was defined by significant linear and quadratic growth, with participants’ PTS levels on average decreasing between W1 and W2 and increasing between W2 and W3, and scores exceeding the cut-off for probable PTSD at each wave.

**Table 3:** Descriptive statistics on posttraumatic stress by most likely class membership, and growth parameters for each trajectory (*N* = 473)

	Low	Decreasing	Increasing	High
	*n* = 322	*n* = 82	*n* = 38	*n* = 31
Posttraumatic Stress				
Wave 1	26.60 (7.01)	53.53 (7.81)	37.85 (8.68)	70.06 (10.36)
Wave 2	25.69 (8.54)	30.74 (8.14)	53.13 (13.29)	52.94 (13.79)
Wave 3	24.69 (7.49)	32.51 (9.06)	54.47 (12.28)	60.94 (8.60)
Growth Parameters				
Linear slope	−0.87 (1.80)	−33.76 (4.05)***	19.24 (8.19)*	−30.69 (9.24)**
Quadratic slope	−0.04 (0.88)	11.73 (1.70)***	−5.56 (3.53)	12.77 (3.97)**

Standard deviations (for posttraumatic stress) and standard errors (for growth parameters) listed in parentheses.

^*^P <* *0.05, ^*^^*^P <* *0.01, ^*^^*^^*^P <* *0.001.

### *RORA* main effects

Results of the three-step analyses testing main effects of *RORA* SNPs on trajectory membership, controlling for age, gender, and ancestry, are shown in Table 4. For *RORA* SNP rs16942660, having one or more copies of the minor allele was significantly associated with a greater likelihood of being in the *increasing* trajectory, relative to the *decreasing* trajectory. However, this result did not hold after correction for multiple testing. In contrast, for rs893290, having one or more copies of the minor allele was associated with a greater likelihood of being in the *high* trajectory, relative to the *low* trajectory, a result that remained significant after correction for multiple testing. The results indicated that each copy of the minor allele is associated with a 3.64 greater odds of being in the *high* trajectory, versus the *low* trajectory. Cross-tabulations by most likelihood class membership (Table 5) found that MAF among participants in the *low* trajectory was 17.1%, compared to 41.9% in the *high* trajectory.

**Table 4:** Main effects and interactions with childhood physical abuse for RORA SNPs on posttraumatic stress trajectory class membership (*N* = 473)

SNP (minor allele)	HI versus LO	INC versus LO	DEC versus LO	HI versus DEC	INC versus DEC	HI versus INC
	OR	OR	OR	OR	OR	OR
Main effects						
rs16942660 (A)	0.15 (0.01, 2.50)	1.80 (0.81, 4.01)	0.58 (0.25, 1.32)	0.26 (0.01, 5.11)	3.13 (1.02, 9.58)*	0.08 (<0.01, 1.69)
rs893290 (C)	3.64 (1.48, 7.65)**	0.47 (0.04, 6.19)	1.54 (0.77, 6.19)	2.18 (0.80, 5.93)	0.30 (0.02, 4.59)	7.20 (0.48, 107.43)
Interaction effects						
rs16942660 (A)	0.99 (0.39, 2.52)	1.18 (0.99, 1.41)	1.15 (0.95, 1.40)	0.85 (0.33, 2.22)	1.02 (0.81, 1.29)	0.83 (0.32, 2.19)
rs893290 (C)	1.32 (1.06, 1.63)*	1.46 (1.03, 2.07)*	1.22 (1.03, 1.45)*	1.07 (0.86, 1.34)	1.19 (0.83, 1.71)	0.90 (0.60, 1.35)

Analyses controlled for age, gender, and ancestry.

HI, High; LO, Low; INC, Increasing; DEC, Decreasing.

Results in boldface held after a Bonferroni-correction of *α*/2 (*P *<* *0.025). ^*^P <* *0.05, ^*^^*^P <* *0.01.

**Table 5:** Percentage of participants in each posttraumatic stress trajectory with different allelic frequencies for RORA SNPs (*N* = 473)

SNP (minor allele)	rs16942660 (A)			rs 893290 (C)		
Latent class	0	1	2	0	1	2
Low (*n *=* *322)	78.0	20.8	1.2	82.3	17.4	0.3
Decreasing (*n *=* *82)	84.1	20.7	2.4	76.8	20.7	2.4
Increasing (*n *=* *38)	71.1	23.7	5.3	89.5	7.9	2.6
High (*n *=* *31)	93.5	6.5	0.0	58.1	41.9	0.0

Genotype designation (0, 1, 2) reflects copies of the minor allele.

### Interactions with childhood physical abuse

**Figure 2:**
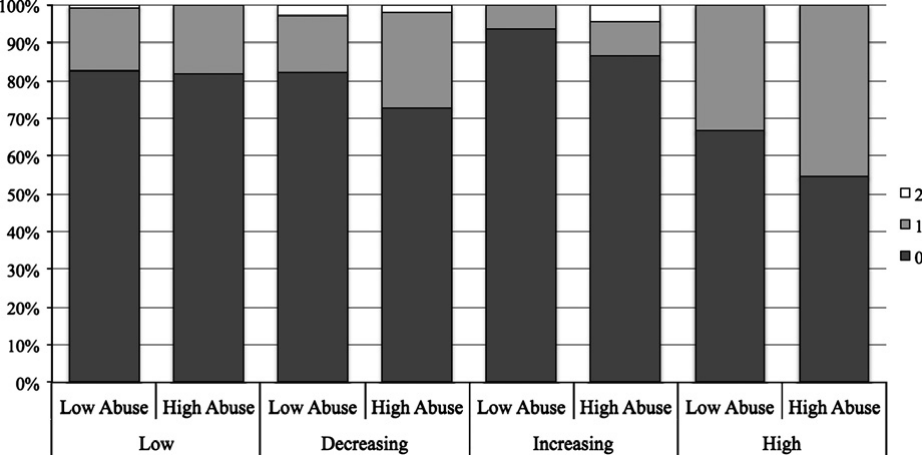
Decomposition of the interaction between childhood physical abuse history and rs893290 on posttraumatic stress trajectory membership. Genotype designation (0, 1, 2) reflects copies of the minor allele.

Table 4 also shows the results of the three-step analyses including interaction effects between *RORA* SNPs and childhood physical abuse. The interaction between rs16942660 and childhood physical abuse was a non-significant predictor of trajectory membership. In contrast, the interaction between rs893290 and childhood physical abuse was significantly predictive of membership in the *high*, *increasing*, and *decreasing* trajectories, all relative to the *low* trajectory. With correction for multiple testing, the interaction remained a significant predictor of *high* and *decreasing* trajectories, relative to the *low* trajectory. Table 6 shows the cross-tabulations between rs893260 and most likely class membership, stratified by level of childhood physical abuse. These values are further illustrated in Fig.2. The pattern of results showed that, among participants with a history of high childhood physical abuse, having one or more copies of the rs893260 minor allele was more strongly associated with membership in the *decreasing* and *high* trajectories, relative to the *low* trajectory, than among participants with a lower history of childhood physical abuse.

**Table 6:** Percentage of participants with rs893260 genotypes within each posttraumatic stress trajectory as a function of childhood physical abuse history (*N* = 473)

Latent Class	Low abuse			High abuse		
	0	1	2	0	1	2
Low (*n *=* *322)	82.6	16.9	0.6	81.9	18.1	0.0
Decreasing (*n *=* *82)	82.4	14.7	2.9	72.9	25.0	2.1
Increasing (*n *=* *38)	93.8	6.3	0.0	86.4	9.1	4.5
High (*n *=* *31)	66.7	33.3	0.0	54.5	45.5	0.0

Genotype designation (0, 1, 2) reflects copies of the minor allele (C). For illustrative purposes, low and high childhood physical abuse were derived through median split.

## Discussion

The results of the study provide preliminary evidence that variation in the *RORA* gene is associated with membership in trajectories of higher PTS and that these associations are stronger among persons exposed to childhood physical abuse. *RORA* SNP rs16942660 was a nominally significant predictor of the *increasing* trajectories, versus the *decreasing* trajectory; however, this finding did not hold after correction for multiple testing. In contrast, *RORA* SNP rs893260 was significantly predictive of the *high* trajectory, versus the *low* trajectory, even after correction for multiple testing. The interaction for rs893260 was statistically predictive of the *high* and *decreasing* trajectories, and nominally predictive of the *increasing* trajectory, relative to the *low* trajectory. Decomposition of the interaction showed that MAF was more strongly associated with membership in each non-*low* trajectory for participants with higher levels of childhood physical abuse.

The results of the study align with those of previous investigations suggesting that variants of the *RORA* gene are associated with PTSD (Logue et al. 2010; Amstadter et al. 2013). Additionally, they suggest that *RORA* may influence both the incidence of PTSD and the course of PTS symptoms over time. Specifically, the main effect of rs893260 suggests that *RORA* might increase risk for chronically elevated symptoms, relative to consistently low symptoms, but not for other symptom trajectories wherein elevations are temporary, either following a delayed or remitting course. On the other hand, the nominally significant effect of rs16942660 could imply that *RORA* increases the likelihood that subthreshold levels of PTS become full-blown PTSD, rather than remit, over time.

Of course, the main effects detected in the analyses should be interpreted in light of the significant interaction effect between rs893260 and childhood physical abuse history in predicting trajectory membership. In our decomposition of the interaction, the evidence suggested that, for participants with histories of lower childhood physical abuse, having one or more copies of the minor allele was only associated with a greater likelihood in the *high* trajectory, relative to the *low* trajectory. However, for those who had experienced more severe childhood physical abuse, one or more copies of the minor allele increased the likelihood of membership in trajectories with any symptom elevations over the course of the study, relative to the *low* trajectory. This is consistent with previous research showing that early life stress enhances the vulnerability associated with risk variants from PTSD candidate genes (Binder et al. 2008; Xie et al. 2009; Nugent et al. 2011). By including trajectories as the dependent variable, we show that risk variants, at least in the case of rs893260, are associated with decreased likelihood of a trajectory of consistently few or no PTS symptoms in the aftermath of trauma, which some might label resilience (Bonanno and Diminich 2013). The interaction observed in the study should be interpreted with caution, however, given the possibility of gene-environment correlation. Although the rGE between rs893260 and childhood physical abuse was nonsignificant (*P *=* *0.31), this could have been due to the relatively small sample size and limited statistical power. The presence of a gene-environment correlation could have led to a spurious finding, or otherwise rendered the interaction uninterpretable (Moffitt et al. 2005; Dick 2011; Nugent et al. 2011). As such, replication of the results with a larger sample is needed.

To understand the biological mechanisms underlying the associations observed in this study, functional data linking *RORA* to PTS is also needed. Although no such data has been published, several potential mechanisms have been posited. For example, Miller and colleagues (Miller et al. 2013) referenced evidence of the role of *RORA* in protection against neurodegeneration in the face of oxidative stress (Jolly et al. 2012). They hypothesized that *RORA* could modify neurodegeneration and functional abnormalities in areas of the brain associated with PTSD and related psychopathology. The authors also proposed alternative biological mechanisms, such as the involvement of *RORA* in processes related to circadian rhythms and steroid hormones that, in turn, have been shown to be dysregulated in cases of PTSD (Jetten 2009; Rasmusson et al. 2010; Germain 2013; Miller et al. 2013).

The results of this study should be interpreted in light of additional limitations. First, the small sample size limited our power to detect significant effects on the one hand, and could have led to spurious findings on the other (Duncan and Keller 2011). This is an important limitation given the small size of some of the trajectories, particularly the *increasing* and *high* trajectories, which were comprised of less than forty participants, and again points to the need for replication. Second, PTS was measured in relation to a heterogeneous array of traumatic events, both in terms of their nature and timing. It is certainly possible that the pattern of results could have differed had all participants experienced the same event (e.g., survivors of the same disaster), or had all been assessed immediately after the trauma. At the same time, a strength of this approach is that the events assessed are reflective of the range of trauma experienced by urban residents. Third, our measure of childhood physical abuse was retrospective, and neither prospective nor archival data were available to validate participants’ reports. However, it is worth noting that childhood physical abuse is most often assessed retrospectively and that a recent study found no differences in the extent to which prospective and retrospective reports of childhood maltreatment were associated with adult psychopathology (Scott et al. 2012). Fourth, as acknowledged previously, our assessment was limited to experiences of childhood physical abuse, whereas other forms of early life stress (e.g., sexual abuse, emotional abuse), as well as traumatic events and stressors experienced in adulthood, were not included. Further research is therefore needed to understand whether the trends observed here are unique to childhood physical abuse, or whether they extend to other stressors and traumatic events. Our assessment also included all experiences of childhood physical abuse up to age 18, and further studies could explore whether the strength of GxEs varies by the age at which stressors and traumatic events were experienced. Lastly, the sample was relatively homogeneous, consisting of primarily African Americans from the same metropolitan area, and replication is therefore needed in populations from other ethnicities and geographic regions. On the other hand, the relative homogeneity of the sample, along with our inclusion of ancestry markers, was advantageous given that genetic variants have been associated with different patterns of risk among different ethnic groups and that population stratification can lead to spurious GxE findings (Nugent et al. 2011).

## Conclusions

These limitations notwithstanding, this study represents a novel contribution to the literature in its inclusion of a recently discovered PTSD candidate gene, evidence of a GxE between *RORA* and a well-established environmental risk factor for PTSD, and use of PTS trajectories, rather than cross-section severity scores of PTSD diagnoses, as the dependent variable. The results suggest that *RORA* might increase risk for severe symptoms that follow a chronic course and, for individuals who have experienced childhood physical abuse, also increase risk for both short- and longer term symptom elevations. Further use of trajectories as the dependent variable could provide greater insight into the role of genetic factors in predicting patterns of PTS over time. Additional studies that employ latent growth curve modeling or growth mixture modeling could also further our understanding of genes and GxEs in shaping the course of PTS over time, that is, changes in symptoms within PTS trajectories.
